# Microbial imbalance in inflammatory bowel disease patients at different taxonomic levels

**DOI:** 10.1186/s13099-019-0341-6

**Published:** 2020-01-04

**Authors:** Mohammad Tauqeer Alam, Gregory C. A. Amos, Andrew R. J. Murphy, Simon Murch, Elizabeth M. H. Wellington, Ramesh P. Arasaradnam

**Affiliations:** 1 Warwick Medical School, University of Warwick, Coventry, UK; 2 School of Life Sciences, University of Warwick, Coventry, UK; 3 Department of Gastroenterology, University Hospitals Coventry & Warwickshire NHS Trust, Clifford Bridge Road, Coventry, CV2 2DX UK; 4 School of Life Sciences, University of Coventry, Coventry, UK; 5 Faculty of Life Science, University of Leicester, Leicester, UK; 6 Present Address: G.C.A.A National Institute for Biological Standards and Control (NIBSC), Potters Bar, UK

**Keywords:** Inflammatory bowel disease, Crohn’s disease, Ulcerative colitis, Gut microbiota, Microbial imbalance

## Abstract

**Background:**

Inflammatory bowel disease (IBD), is a debilitating group of chronic diseases including Crohn’s Disease (CD) and ulcerative colitis (UC), which causes inflammation of the gut and affects millions of people worldwide. At different taxonomic levels, the structure of the gut microbiota is significantly altered in IBD patients compared to that of healthy individuals. However, it is unclear how these IBD-affected bacterial groups are related to other common bacteria in the gut, and how they are connected across different disease conditions at the global scale.

**Results:**

In this study, using faecal samples from patients with IBD, we show through diversity analysis of the microbial community structure based on the 16S rRNA gene that the gut microbiome of IBD patients is less diverse compared to healthy individuals. Furthermore, we have identified which bacterial groups change in abundance in both CD and UC compared to healthy controls. A substantial imbalance was observed across four major bacterial phyla including Firmicutes, Bacteroidetes, Proteobacteria and Actinobacteria, which together constitute > 98% of the gut microbiota. Next, we reconstructed a bacterial family co-abundance network based on the correlation of abundance profiles obtained from the public gut microbiome data of > 22,000 samples of faecal and gut biopsies taken from both diseased and healthy individuals. The data was compiled using the EBI metagenomics database (Mitchell et al. in Nucleic Acids Res 46:D726–D735, 2018). By mapping IBD-altered bacterial families to the network, we show that the bacterial families which exhibit an increased abundance in IBD conditions are not well connected to other groups, implying that these families generally do not coexist together with common gut organisms. Whereas, the bacterial families whose abundance is reduced or did not change in IBD conditions compared to healthy conditions are very well connected to other bacterial groups, suggesting they are highly important groups of bacteria in the gut that can coexist with other bacteria across a range of conditions.

**Conclusions:**

IBD patients exhibited a less diverse gut microbiome compared to healthy individuals. Bacterial groups which changed in IBD patients were found to be groups which do not co-exist well with common commensal gut bacteria, whereas bacterial groups which did not change in patients with IBD were found to commonly co-exist with commensal gut microbiota. This gives a potential insight into the dynamics of the gut microbiota in patients with IBD.

## Introduction

Inflammatory bowel disease (IBD), a group of chronic intestinal disorders including Crohn’s disease (CD) and ulcerative colitis (UC), causes inflammation of the gut and affects millions of people worldwide [1–4]. Both CD and UC diseases are differentiated by their location and levels of inflammation in the gastrointestinal (GI) tract. UC mostly involves inflammation to the rectum and colon, whereas CD most often affects the terminal ileum and colon though in some cases it can affect any part of the GI tract [2, 5]. Currently, there is no full cure for IBD, but different treatments such as taking steroids, immunosuppressants, liquid diet or surgery can help in reducing the symptoms [5]. To date, the exact cause of IBD is not understood, however, a combination of genetic variants, environmental factors, deregulated host immune system, and gut microbiota dysbiosis is associated with IBD [6–16].

More than 215 IBD-associated loci have been identified so far from various genome-wide association studies (GWAS) [7]. It has been reported that a large percentage (~ 30%) of these loci are common between CD and UC, showing involvement of common biological processes in both conditions [14, 17]. Moreover, these IBD-associated loci are mostly involved with immune system deregulation, a process which the gut microbiome has also been implicated in [18]. The gut microbiota, which has a complex community of a hundred trillion bacterial and archaeal cells comprising more than a thousand species, provides benefits to the host such as short-chain fatty acids (SCFA) and amino acids, metabolism of undigested carbohydrate, and stimulation of the immune system [19, 20]. In patients with IBD, the structure and the composition of the gut microbiota is severely altered compared to that of a healthy condition [10, 13, 18, 21]. Previous work has reported imbalances in IBD patients for the Firmicutes and Bacteroidetes at the phylum level, and Ruminococcaceae, Veillonellaceae, Christensenellaceae, Bacteroidaceae, and Rikenellaceae at the family level. However, there is a large degree of variability across studies, with many reporting contradictory findings. In particular, it is unclear what the relationship is between microbial groups when there is inflammation of the gut epithelium during various diseased states. There is furthermore, a general knowledge gap in establishing the relationships between microbial groups across different disease conditions.

The aim of the current work was to investigate the relationships between changing microbial groups in IBD. In particular, we wanted to understand which microbial groups differ during IBD, and how these groups differ in co-abundance patterns across a variety of diseases at the global scale. To do this, we initially investigated the gut microbial imbalance, at different taxonomic levels for healthy volunteers and CD and UC patients. We next reconstructed a network of the co-abundance patterns of different bacterial groups using publicly available data from a variety of studies at a global scale. Our results indicate that the bacterial groups which increase in abundance during IBD are specific to both CD and UC conditions. In comparison, bacterial groups which did not change in abundance during different disease states are well connected in global networks, giving us a better understanding of the dynamics of the microbiome in both health and disease.

## Results and discussion

### Gut microbial richness in IBD patients

**Fig. 1:**
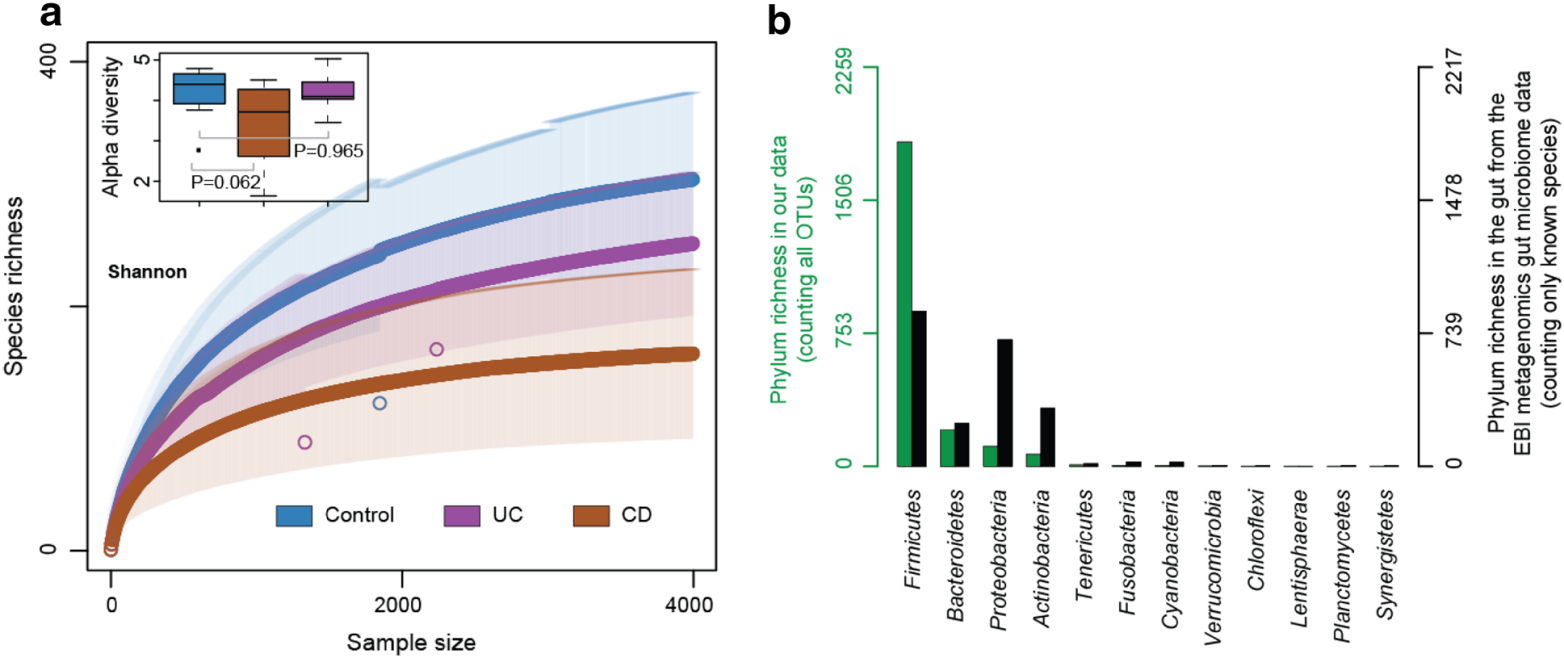
Microbial diversity and richness. **a** Species richness is substantially less in CD patient samples compared to the healthy control and UC patient samples. Shannon alpha diversity plot demonstrate that CD patients samples are less diverse compared to the healthy control and UC patient samples (inset figure). **b** Phylum level richness in the gut microbiota from our samples (left side Y-axis shown in green) compared to the global gut microbial species richness obtained across more than 20 K samples from a variety of conditions (right side Y-axis shown in black)

We collected faecal samples from 30 individuals (9 CD patients, 11 UC patients and 10 healthy volunteers) and performed 16S rRNA taxonomic profiling to understand changes in community structure during disease with resulting data analysed using the DADA2 pipeline. Amplicon sequence variants (ASVs) were used as a measure of diversity. As previously reported, species richness in the gut of IBD patients (both CD and UC) was lower than that of healthy volunteers [10, 13]. Moreover, within the IBD patients groups, the gut of CD patients exhibit substantially lower species richness than that of UC patients (Fig. 1a). As a measure of diversity, we identified a total of 2261 ASVs, of which 81% belong to the Firmicutes, 9.25% Bacteroidetes, 5.13% Proteobacteria, and 3.14% ASVs are from Actinobacteria. Combined, these four phyla constitute more than 98% of the total identified ASVs [22, 23]. To understand how this compared to other studies, we investigated the global microbial species-richness in the human gut across > 22,000 samples from 113 different studies from a variety of conditions (EBI metagenomics accession numbers [1] in Additional file 1: Table S1). We have considered only the known species in each study and made a unique list of gut bacterial species across studies. Similar to our experimental observations, the four phyla including Firmicutes, Bacteroidetes, Proteobacteria and Actinobacteria comprised > 94% of the total diversity in the gut, with the Firmicutes being the most species-rich phylum across conditions (Fig. 1b). However, the number of species identified as belonging to the Firmicute Phyla in our dataset (81% of all identified species from 20 IBD and 10 healthy condition samples) is substantially higher than what is usually reported at a global level (38.18%). As the observation comes from 2/3 of IBD patient and 1/3 of healthy control samples, this increased level of Firmicutes species-richness was attributed to the high number of IBD samples. Analysis of global studies for species-richness of Proteobacteria demonstrated this phyla usually accounts for 31.18% of all species, however this was substantially lower in our dataset (5.13%). Finally, the richness of Bacteroidetes was also reduced in our dataset compared to the global datasets (9.24% in our study compared to 14.35% globally). We find a similar observation when considering all OTUs from individual EBI gut microbiome studies (Additional file 2: Figure S1).

After identifying the differences at different taxonomic levels for each disease condition, for future work it is crucial to understand the reasons for such dysbiosis and whether they are causative or consequential of disease. Studies suggest that metabolic dependency [24] and nutritional preferences [25] between microorganisms are driving forces in microbial community formation. For example, metabolic cooperation between bacteria is crucial to microbial assemblages and changes to this could cause shifts across the whole community. For future work, it would be interesting to investigate the microbial metabolic interactions during disease and how this compares to a healthy gut.

### The gut microbial abundance at different taxonomic levels in IBD patients

**Fig. 2:**
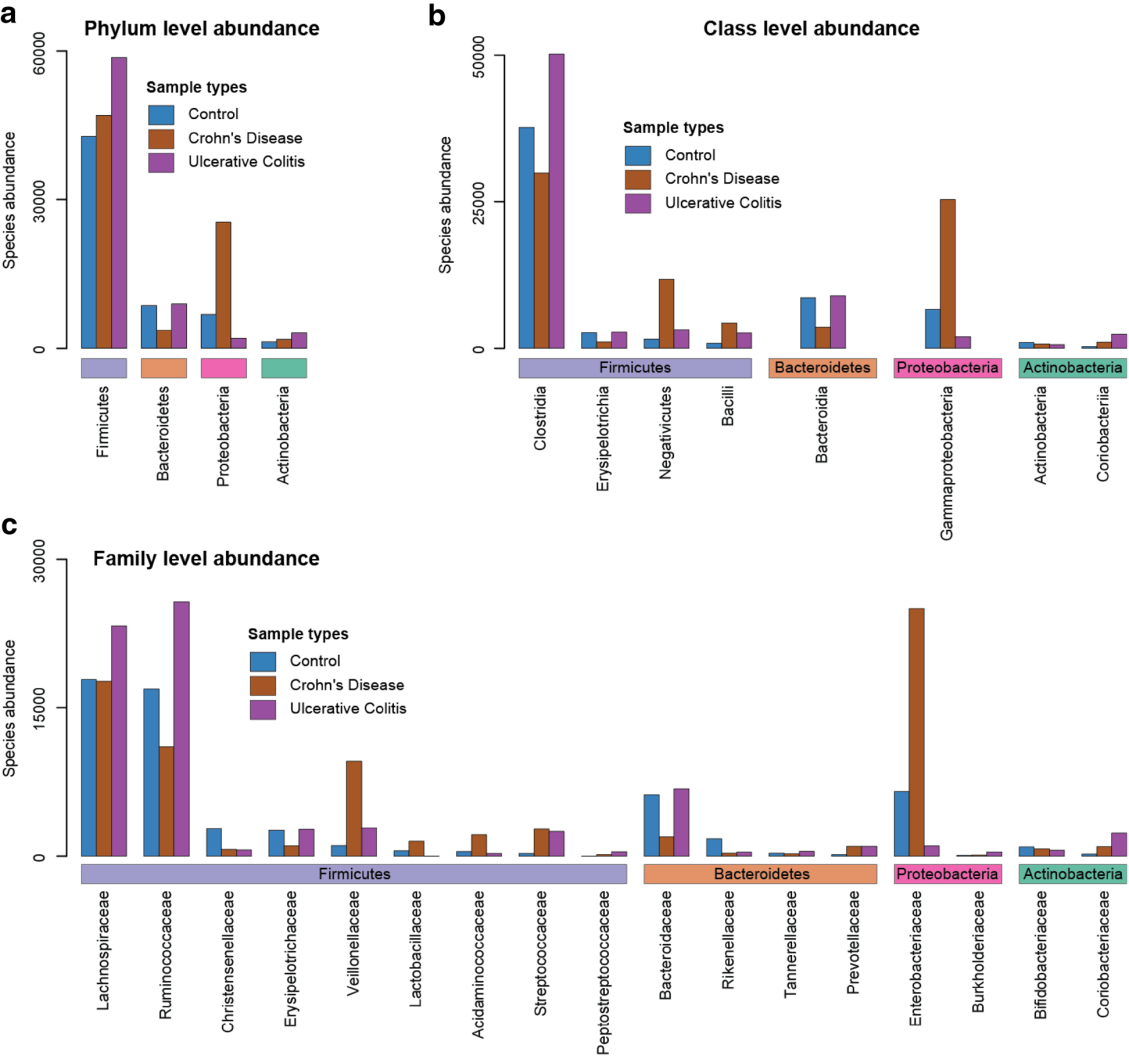
The gut microbial abundance. **a** Phylum, **b** Class and **c** Family level abundance in different conditions. Classes and families belonging to the four most abundant phylum across conditions are grouped according to phylum

Compared to the healthy controls, both IBD patient groups (CD and UC patients) demonstrated strong microbial imbalance at different taxonomic levels (Fig. 2). At the phylum level, both IBD conditions exhibit an increased abundance of Firmicutes and Actinobacteria, relative to the controls. In particular, the abundance of this phyla during UC, was far greater than CD or healthy controls. For the two other dominant bacterial phyla, the Proteobacteria and Bacteroidetes, the abundance profiles varied across disease conditions (Fig. 2a). In CD, the abundance of Bacteroidetes, which is often associated with a healthy gut, was deceased 2.4-fold, whereas the abundance of Proteobacteria, a phyla associated including wide variety of pathogens, was increased 3.8 fold. Interestingly, for patients with UC the abundance of Proteobacteria was decreased (3.4 fold) relative to controls and there was no significant differences in levels Bacteroidetes [26]. Several studies have reported the gut microbial imbalances for IBD, however, the imbalance at the level of different phylum is variable across studies [10, 13, 18, 21, 27, 28]. This could likely be a reflection on the lack of standardisation across microbiome techniques, or perhaps due to a heterogeneity in the microbiome associated with disease.

We further investigated how different taxonomic levels belonging to each of the main four phyla, Firmicutes, Proteobacteria, Bacteroidetes, and Actinobacteria, were changed during IBD. For Firmicutes, the most abundant phylum in the gut in all conditions, we observed four classes and nine different families which changed in abundance relative to healthy controls. For CD patients, the abundance of two classes including Clostridia and Erysipelotrichia, was reduced, and three families including Ruminococcaceae, Christensenellaceae and Erysipelotrichaceae were reduced relative to healthy controls. The level of two other two classes such as Negativicutes and Bacilli (obligately aerobic) and five families including Veillonellaceae, Lactobacillaceae, Acidaminococcaceae, Streptococcaceae and Peptostreptococcaceae was increased, similar to the imbalance in their parent phylum Firmicutes. Interestingly, Lachnospiraceae, the most abundant Firmicutes family, was at a similar level to the control. For UC patients, the abundance of the Firmicute classes Clostridia, Negativicutes and Bacilli, and Firmicute families Ruminococcaceae, Lachnospiraceae, Veillonellaceae, Streptococcaceae and Peptostreptococcaceae were increased. The Erysipelotrichia class and Erysipelotrichaceae family were the same as the controls, whereas, three families including Acidaminococcaceae, Christensenellaceae and Lactobacillaceae, were reduced in abundance. For Bacteroidetes, which is the only reduced phylum in CD, we observed a reduced abundance in the Bactersoidia class and Bacteroidia families Bacteroidaceae and Rikenellaceae. The Prevotellaceae family, in particular, was increased in CD patients. For UC patients, the only families to change of the Bacteroidetes was the Rikenellaceae and Tannerellaceae families which were decreased in abundance, and the Prevotellaceae which increased in abundance as with CD. Finally, for the Proteobacteria phylum, we observed an imbalance in Enterobacteriaceae and Burkholderiaceae families, with the abundance level of Enterobacteriaceae increased in CD patients and decreased in UC patients compared to the controls. Burkholderiaceae abundance was increased for both CD and UC patients. Finally, for the Actinobacteria phylum, the abundance level of both the class Coriobacteriia and family Coriobacteriaceae was increased in both IBD conditions relative to the controls, whereas the class Actinobacteria and family Bifidobacteriaceae was reduced [13, 29]. In summary, we demonstrate that multiple families of a class, classes of a phyla differ between both the IBD conditions, and between each IBD condition and healthy control. This suggests that changes in one bacterial family has consequences for others. To investigate this further, we used co-occurrence network analysis to establish patterns of how bacterial groups increase and decrease across global studies.

### Global co-abundance in the gut of different bacterial families

**Fig. 3:**
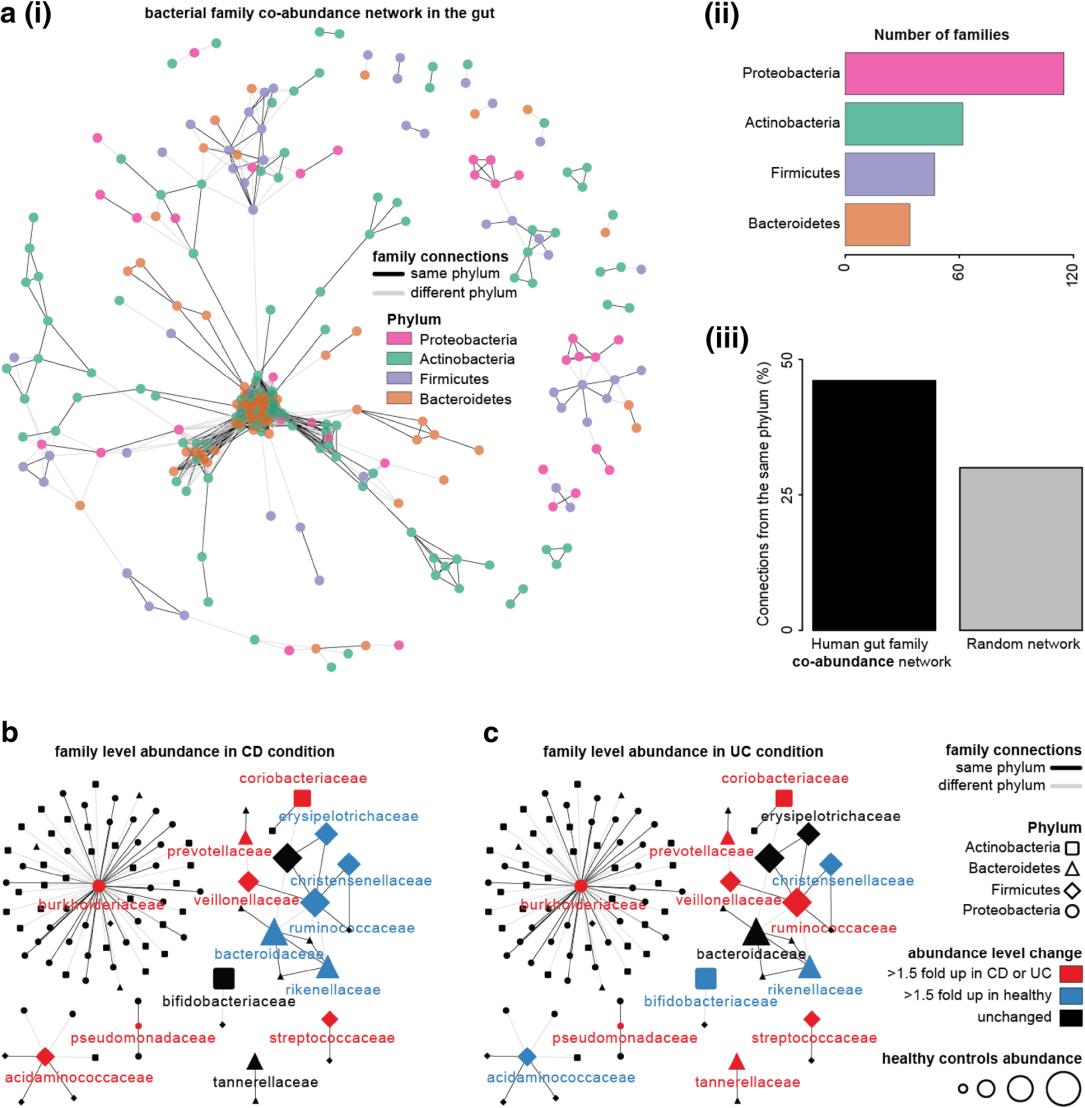
The human gut bacteria family co-abundance. **a**, **i** The network represents the global human gut bacterial family coexistence considering the four most highly abundant gut bacterial phyla. The network connections are based on correlation test (P-value < 1e−10 and Pearson’s correlation coefficient > 0.3). Edge connection between families from the same phylum is shown in black lines whereas the family connection between different phyla is shown in grey lines. Family nodes are coloured according to phylum. **a**, **ii** This graph demonstrates the number of families (i.e. nodes) belonging to a different phylum in the global bacterial family coexistence. **a**, **iii** The percentage connection between families from the same phylum is substantially higher in the global bacterial family coexistence compared to a random network of the same size. **b** A subnetwork of the global human gut bacterial family coexistence network where the abundance level of at least one family node in a connection is > 1.5-fold higher in either CD or healthy condition against each other. **c** Similarly, a subnetwork of the global human gut bacterial family coexistence network where the abundance level of at least one family node in a connection is > 1.5 fold higher in either UC or healthy condition against each other. Edge connection between families from the same phylum is shown in black lines whereas the family connection between different phylum is shown in grey lines. Family belonging to different phyla are shown in different shapes. The node colour shows the increased abundance level in a disease (CD or UC) or healthy condition compared to each other. The size of the node represents the abundance level in a healthy condition

The gut microbiota abundance profiles from > 22,000 samples across a variety of conditions from 113 different studies was analysed to explore how different bacterial groups change across global studies. Using the Pearson’s correlation test, we built a network of significantly co-abundant (Pearson’s correlation coefficient > 0.3 and *P* value < 1e−10) bacterial families across a range of conditions collected from global studies (Fig. 3ai). We observed that the majority of bacterial families in the network belonged to the phyla Proteobacteria, Actinobacteria, Firmicutes and Bacteroidetes (Fig. 3aii). In the global gut bacterial family–family co-abundance network (Fig. 3ai), family nodes of 45% of the total connections are from the same phylum, compared to a random network of the same size where percentage connections were much lower (~ 30%) (Fig. 3aiii). This suggests that groups from the same phylum, which likely have similar metabolic requirements, are likely to change abundance as a collective. To understand the family level microbial imbalance during IBD, we further considered a subnetwork of the global family co-abundance network, where at least one family node was from the most abundant bacterial families in either CD, UC or healthy subjects. In this subnetwork, we highlighted bacterial families which were changed during CD or UC, compared to the healthy (Fig. 3b, c).

In CD, seven bacterial families including Coriobacteriaceae, Prevotellaceae, Burkholderiaceae, Veillonellaceae, Streptococcaceae, Pseudomonadaceae and Acidaminococcaceae have increased abundance compared to the healthy controls (Fig. 2c), however, only two families including Prevotellaceae and Veillonellaceae are connected in the global network (Fig. 3b). In contrast, the level of five other families including Erysipelotrichaceae, Christensenellaceae, Ruminococcaceae, Bacteroidaceae and Rikenellaceae, were well connected in the global network (Fig. 3b) and had reduced abundance in CD (Fig. 2c). Similarly, for UC, families with an increased abundance in UC were less well connected on the global scale (Fig. 3c). This suggests that bacterial groups which increase in abundance during IBD, are not typically associated with the healthy gut microbiome, nor do they commonly co-exist with commensals observed in the healthy gut. Furthermore, families which had increased abundance levels in healthy conditions compared to CD are very well connected, suggesting microbes in the gut of healthy individuals exist as a co-operative microbial assemblage. In particular, the connection between families such as Bacteroidaceae and Ruminococcaceae in the co-abundance network indicates that they may coexist together in across conditions, potentially due to similarities in physiology or the presence of metabolically cooperating species. For future work, it would be highly interesting to examine species of these families and investigate the relationships between these organisms.

## Conclusions

In summary, our analysis demonstrates that IBD patients (both CD and UC) and healthy volunteers have reduced species richness, and imbalances in families, classes, and phyla, relative to healthy volunteers. Four bacterial phyla including Firmicutes, Bacteroidetes, Proteobacteria and Actinobacteria comprised > 98% of the species in this study. To understand how bacteria assemblages depend on co-operation, we reconstructed a large co-abundance network based on the public gut microbiome data of > 22,000 samples. From this we demonstrated that the bacterial families which have an increased abundance level in IBD conditions are not well connected to other bacterial groups in the global family co-abundance network. This suggests that these bacteria do not co-exist with healthy gut microbial commensals and supports the concept that healthy assemblages are dependent on metabolic co-operation, due to the high connectivity of bacterial groups found in healthy conditions across > 22,000 samples.

## Methodology

### Sample collection and DNA extraction

Samples were collected from patients in standard 300 ml sterilin tubes and frozen immediately in −80 °C. Patients were asked to produce the first-morning sample for consistency and to avoid alcohol the previous 24 h. Samples were thawed and DNA was extracted using FastDNA Spin Kit for Soil (MPBiomedicals) [30] as per the manufacturer’s instructions.

## 16S rRNA sequencing

454 pyrosequencing using 16S universal eubacterial primers 27F and 534R [31] was performed by Molecular Research (MRDNA), Shallowater, Texas, using an adapted protocol developed in [32]. Number of reads per sample ranged from 6936 to 100,972, with an average of 38,931 reads per sample.

### Bioinformatic analysis of 16S rRNA sequencing data

16S rRNA high-throughput sequencing data was analysed by following the workflow from Callahan et al. [33]. Quality checking, filtering and trimming of fastq files were performed by functions from the dada2 package in R [34]. After filtering the reads, high-resolution Amplicon Sequence Variants (ASVs) were inferred using dada function [34]. ASVs are a higher-resolution analogue of the traditional OTUs. Chimeric sequences were removed and taxonomy assigned to ASVs based on the naive Bayesian classifier method with silva_nr_v132_train_set.fa as the training set [34]. Species-richness and alpha diversity (Shannon) were analysed by plot_richness function from the phyloseq package in R [35]. To make the rarefaction species richness curve, ‘rarecurve’ function from the vegan package [36] in R was used.

### Statistical analysis: bacterial family co-abundance network based on microbiome data

Taxonomic assignments, containing a detailed taxonomy and abundance data of OTUs or ASVs in samples, of 113 gut microbiome studies, covering more than 22,000 samples, were downloaded from the EBI metagenomics database [1]. The data were then parsed and tables containing bacterial abundance from different phyla, classes and families were generated. Abundance at the level of phylum was then used for the global gut microbial abundance. The family-level bacterial abundance was used to construct the bacterial family–family coexistence network. For each pair of bacterial families, Pearson’s correlation test was performed. Family nodes were connected when P-value < 1e−10 and Pearson’s correlation coefficient > 0.3.

## Supplementary information

**Additional file 1: Table S1.** List of gut microbiome studies from EBI metagenomics database.

**Additional file 2: Figure S1.** Phylum level richness in the gut microbiota from our samples (shown in green) compared to the gut microbial species richness obtained across studies, each with more than 100 OTUs (total 81 study), from a variety of conditions (shown in black).

## Data Availability

Sequence data of the study has been submitted to European Nucleotide Archive (ENA) (https://www.ebi.ac.uk/ena/submit/sra/#studies) under the Accession Number PRJEB33711
