# Supplementary material for: Microbial imbalance in inflammatory bowel disease patients at different taxonomic levels
Source: Gut Pathog. 2020 Jan 4;12:1. doi: 10.1186/s13099-019-0341-6 (PMC6942256; doi:10.1186/s13099-019-0341-6)
Supplement: Supplementary file 1 — Additional file 1: Table S1. List of gut microbiome studies from EBI metagenomics database. [file 13099_2019_341_MOESM1_ESM.docx]

**Additional file 1: Table S1.** List of gut microbiome studies from EBI metagenomics database.

| **Study ID (EBI)** | **Study Name** | **NCBI Project ID** |
| --- | --- | --- |
| ERP024243 | 16S data - Multi-omics Differentially Classify Disease State and Treatment Outcome in Pediatric Crohn's Disease | PRJEB21933 |
| ERP105247 | 16S data from FMT trial for the treatment of recurrent Clostridium difficile infection | PRJEB23489 |
| ERP019639 | 16S rRNA gene sequencing in extremely low birth weight infant gut | PRJEB17754 |
| ERP019673 | 16S rRNA gene sequencing of Extremelly low birth weight infant | PRJEB17783 |
| ERP019785 | 16S rRNA gene sequencing of the extremelly birth weight infant gut | PRJEB17882 |
| SRP055567 | 16S rRNA profiling of "Allergy" and "Healthy" human faecal samples | PRJNA276631 |
| SRP066323 | 16s-based microbiome metagenome | PRJNA302437 |
| SRP000319 | A core gut microbiome in obese and lean twins | PRJNA32089 |
| ERP000108 | A human gut microbial gene catalog established by deep metagenomic sequencing (MetaHIT) | PRJEB2054 |
| SRP076281 | A Longitudinal Study of Pediatric Subjects with Newly Diagnosed Inflammatory Bowel Disease | PRJNA324825 |
| ERP002061 | A method for identifying metagenomic species and variable genetic elements by exhaustive co-abundance binning | PRJEB1220 |
| ERP004264 | Alterations of the Fecal Microbiome in Parkinson's Disease | PRJEB4927 |
| SRP075039 | Alterations of the human gut microbiome in multiple sclerosis | PRJNA321051 |
| ERP021378 | Analysis of stool samples from sickle cell disease patients and healthy controls | PRJEB19367 |
| SRP115612 | Association between oral and gut microbiota in Periodontal Disease | PRJNA396275 |
| ERP001506 | Beta Lactam Antibiotics and Human Gut Microbiota | PRJEB3085 |
| SRP008047 | BGI Type 2 Diabetes study |  |
| SRP070464 | Capsule Patients | PRJNA311224 |
| ERP008562 | Changes imposed by Clostridium difficile infection on the human gut microbiome | PRJEB7637 |
| SRP070699 | Colonic transit time relates to bacterial metabolism and mucosal turnover in the gut | PRJNA306419 |
| ERP009244 | Comparison of distal gut microbiota structure and function in US and Egyptian children | PRJEB8201 |
| SRP073722 | Comparison of the fecal microbiota during variable collection and storage methods | PRJNA317493 |
| ERP001706 | Crohn's Disease viral and microbial metagenome | PRJEB3206 |
| SRP002437 | Developing infant gut microbiome |  |
| ERP001956 | Diagnostic Metagenomics: A Culture-Independent Approach to the Investigation of Bacterial Infections | PRJEB1775 |
| ERP013102 | Dietary intervention with a probiotic Bifidobacterium modulates dominant bacterial taxa in an enterotype-dependent fashion and normalizes butyrate levels in the gut of healthy adults | PRJEB11694 |
| SRP073172 | DNA from FIT can replace stool for microbiota-based colorectal | PRJNA318004 |
| ERP005989 | Dynamics and Stabilization of the Human Gut Microbiome during the First Year of Life | PRJEB6456 |
| ERP015450 | Dysbiosis of gut microbiota contributes to the pathogenesis of hypertension | PRJEB13870 |
| SRP013615 | Effect of oral immunization with the Ty21a typhoid vaccine on local and systemic immune responses and the gut microbiota in adults | PRJNA167398 |
| ERP106248 | Effect of Yoga and low-FODMAP diet in the intestinal microbiota of patients with IBS | PRJEB24421 |
| ERP005883 | Effects of cholera on the human gut microbiota, and interactions between human gut microbes and Vibrio cholerae. | PRJEB6358 |
| ERP106358 | EMG produced TPA metagenomics assembly of the Compositional_dynamics_of_intestinal_spore_forming_bacteria (Compositional_dynamics_of_intestinal_spore_forming_bacteria) data set. | PRJEB24528 |
| ERP104210 | EMG produced TPA metagenomics assembly of the DIPP Diabetes Microbiome (DIPP Diabetes Microbiome) data set | PRJEB22529 |
| ERP104039 | EMG produced TPA metagenomics assembly of the Dynamics and Stabilization of the Human Gut Microbiome during the First Year of Life (InfantGut) data set | PRJEB22360 |
| ERP104230 | EMG produced TPA metagenomics assembly of the Evaluation of vertical transmission between mother-infant (human gut metagenome) data set | PRJEB22549 |
| ERP104223 | EMG produced TPA metagenomics assembly of the Gut metagenome from two premature infants (human gut metagenome) data set | PRJEB22542 |
| ERP104182 | EMG produced TPA metagenomics assembly of the Gut microbial metabolism shifts towards a more toxic profile with supplementary iron in a kinetic model of the human large intestine (TIM2_iron_study) data set | PRJEB22501 |
| ERP104228 | EMG produced TPA metagenomics assembly of the Homo sapiens fecal metagenome Raw sequence reads (human gut metagenome) data set | PRJEB22547 |
| ERP104233 | EMG produced TPA metagenomics assembly of the Human Gut Microbiome Metagenome (human gut metagenome) data set | PRJEB22552 |
| ERP104235 | EMG produced TPA metagenomics assembly of the Increased intestinal microbial diversity following fecal microbiota transplant for active Crohn''s disease (human gut metagenome) data set | PRJEB22554 |
| ERP106359 | EMG produced TPA metagenomics assembly of the Liver cirrhosis occurs as a consequence of many chronic liver diseases that are prevalent worldwide. | PRJEB24529 |
| ERP104179 | EMG produced TPA metagenomics assembly of the metagenome fecal microbiota, Ilumina seq reads of 12 individuals at 2 timepoints (EKmeta) data set | PRJEB22498 |
| ERP104071 | EMG produced TPA metagenomics assembly of the Metagenome sequencing of the Hadza hunter-gatherer gut microbiota. (human gut metagenome) data set | PRJEB22391 |
| ERP106364 | EMG produced TPA metagenomics assembly of the Metagenomes from human infant fecal samples with and without necrotizing enterocolitis (human gut metagenome) data set. | PRJEB24534 |
| ERP104072 | EMG produced TPA metagenomics assembly of the Metagenomic analysis of infant stool sample (human gut metagenome) data set | PRJEB22392 |
| ERP104209 | EMG produced TPA metagenomics assembly of the Microbial composition of samples from infant gut (human gut metagenome) data set | PRJEB22528 |
| ERP104044 | EMG produced TPA metagenomics assembly of the Pediatric Fecal Metagenome (human gut metagenome) data set | PRJEB22365 |
| ERP104219 | EMG produced TPA metagenomics assembly of the Rapid evolution of the human gut virome (human gut metagenome) data set | PRJEB22538 |
| ERP104200 | EMG produced TPA metagenomics assembly of the Roux-en-Y gastric bypass surgery of morbidly obese patients shows swift and persistent changes of the individual gut microbiota (GBP) data set | PRJEB22519 |
| ERP106171 | EMG produced TPA metagenomics assembly of the The antibiotic resistance potential of the preterm infant gut microbiome measured using shotgun metagenomics. (Antibiotic resistance within the preterm infant gut.) data set. | PRJEB24355 |
| ERP104186 | EMG produced TPA metagenomics assembly of the The initial state of the human gut microbiome determines its reshaping by antibiotics (Impact of cefprozil on the gut microbiome of healthy individuals) data set | PRJEB22505 |
| ERP105876 | EMG produced TPA metagenomics assembly of the The microbial colonization of the intestine during the first months of life constitutes the most important process for the microbiota-induced host-homeostasis. | PRJEB24076 |
| ERP020191 | Estimation of variability in the gut microbiota resistome of the Russian citizens aimed at identification of pathways for transmission and spread of antibiotic resistance. | PRJEB18265 |
| ERP008885 | Evidence for salt tolerance in the human gut mobilome and potential mechanisms. | PRJEB7899 |
| SRP045568 | Fecal microbiota of toddlers Metagenome | PRJNA258387 |
| ERP001739 | Follow-up of faecal microbiota in IBS patients | PRJEB3227 |
| ERP015317 | Genetic determinants of the gut microbiome in the TwinsUK cohort | PRJEB13747 |
| ERP002469 | Gut metagenome in European women with normal, impaired and diabetic glucose control | PRJEB1786 |
| ERP006091 | Gut microbial metabolism shifts towards a more toxic profile with supplementary iron in a kinetic model of the human large intestine | PRJEB6542 |
| ERP010229 | Gut microbial succession follows acute secretory diarrhea in humans | PRJEB9150 |
| ERP021093 | Gut microbiome from patients obtained by 16s rRNA sequencing. | PRJEB19103 |
| ERP005185 | Gut microbiota in chronic kidney disease | PRJEB5761 |
| ERP013262 | Gut microbiota of Juvenile Idiopathic arthritis | PRJEB11846 |
| ERP010458 | Gut microbiota of stroke patients differentiates from healthy controls | PRJEB9365 |
| SRP064846 | Homo sapiens fecal microbiome transplant | PRJNA298590 |
| ERP006059 | Host lifestyle affects human microbiota on daily timescales | PRJEB6518 |
| ERP004846 | Human faeces Metagenome | PRJEB5431 |
| SRP001709 | Human fecal microbiome | PRJNA43253 |
| SRP068240 | Human feces metagenome 16s rDNA sequencing | PRJNA307231 |
| SRP073516 | Human Feces Samples Metagenome | PRJNA318788 |
| SRP065497 | Human gut environment Targeted loci environmental | PRJNA280026 |
| SRP076746 | human gut metagenome Metagenome | PRJNA326143 |
| SRP002423 | Human Gut Microbiome in Crohn's Disease | PRJNA46321 |
| ERP005196 | Human gut microbiota associated to Clostridium difficile infection | PRJEB5771 |
| SRP049113 | Human gut microbiota from the ALADDIN study | PRJNA263853 |
| ERP018622 | ICU_metagenomics_Using_16S_rRNA_analysis_for_assessing_the_respiratory_bacterial_infection_threat_to_immunocompromised_patients_within_Intensive_Care_Units | PRJEB16762 |
| ERP013827 | Impact of faecal microbiota transplantation on the intestinal microbiome in metabolic syndrome patients | PRJEB12357 |
| ERP021966 | Impact of the diet on the intestinal microbiota in African children moving from rural to urban areas | PRJEB19895 |
| ERP001596 | Long insert human faecal metagenomic library. | PRJEB3148 |
| ERP022894 | Low FODMAP diet and probiotics in irritable bowel syndrome: a 2x2 factorial design, randomised, placebo-controlled trial | PRJEB20721 |
| SRP067761 | Managing human microbiomes: Explaining heterogeneous responses in butyrate to dietary supplementation with resistant starch | PRJNA306884 |
| ERP005558 | Metagenome fecal microbiota, Illumina seq reads of 12 individuals at 2 timepoints | PRJEB6092 |
| SRP067522 | Metagenome from fecal samples collect from healty human subject during probiotic intervention trial | PRJNA306016 |
| ERP007044 | metagenomic analysis of human gut microbiome | PRJEB7331 |
| SRP012558 | Metagenomic analysis of infant stool sample | PRJNA60717 |
| ERP104944 | Metagenomic characterization of the human intestinal microbiota in faecal samples from STEC-infected patients | PRJEB23207 |
| ERP023970 | Metagenomics 1st 5 data | PRJEB21696 |
| SRP001634 | Microbial composition of samples from infant gut | PRJNA63661 |
| ERP012549 | Microbiota composition in Anorexia Nervosa patients before and after weight gain as compared to healthy controls | PRJEB11199 |
| ERP011526 | Modulation of dyslipidemic children fecal microbial ecosystem by dietary intervention with hazelnuts as source of unsaturated fatty acids. | PRJEB10296 |
| SRP064888 | Modulation of the infant gut microbiota by a starter infant formula containing a synbiotic of bovine milk-derived oligosaccharides and Bifidobacterium animalis subsp. lactis CNCM I-3446 | PRJNA298960 |
| ERP001038 | Mutualism between gut microbiota and the host as revealed in a comparative study of breast-fed versus formula-fed infants | PRJEB2778 |
| ERP012953 | NeoM | PRJEB11554 |
| SRP067629 | Phage therapy in Bangladeshi children hospitalized with acute bacterial diarrhea | PRJNA306615 |
| ERP021080 | Potential and active functions in the gut microbiota of a healthy human cohort | PRJEB19090 |
| ERP023432 | Randomized controlled trial on the impact of early live intervention with bifidobacteria on the healthy infant fecal microbiota and metabolite profile | PRJEB21196 |
| SRP077632 | Raw sequence reads of human sleep apnea patients gut microbiome | PRJNA323360 |
| ERP014628 | Reduced diversity and altered composition of the gut microbiome in individuals with Myalgic Encephalomyelitis/ChronicFatigue Syndrome | PRJEB13092 |
| ERP002222 | Shifts in Human Intestinal Microbiota after Smoking Cessation | PRJEB1421 |
| ERP013496 | Study of early life microbiota in preterm baby | PRJEB12057 |
| ERP012163 | Study of the abundance of bacteria from human samples | PRJEB10865 |
| SRP067039 | Targeted Gene amplicons raw sequence reads | PRJNA305094 |
| ERP016615 | Term and preterm shotgun samples | PRJEB14935 |
| ERP016968 | The antibiotic resistance potential of the preterm infant gut microbiome measured using shotgun metagenomics. | PRJEB15257 |
| ERP007244 | The comparison of gut microbiota obtained from centenarian, elderly, and adults living in southwestern longevity belt in Korea | PRJEB7507 |
| ERP009053 | The Effect of Probiotics with Antibiotics on Gut Microbiota during the Helicobacter Eradication: Randomized Controlled Trial | PRJEB8022 |
| ERP011562 | The Effect of Propidium Monoazide Treatment on Identification of Bacterial Communities of Very Low Birth Weight Preterm Infant Faeces Analysed by 16s rRNA Gene Sequencing | PRJEB10326 |
| ERP008951 | The fecal microbiome was studied in a group of IBD suffers and compared to a control group's fecal microbiome | PRJEB7949 |
| ERP009131 | The initial state of the human gut microbiome determines its reshaping by antibiotics | PRJEB8094 |
| DRP001277 | The prebiotic fructooligosaccharide modulates metabolic dynamics and IgA production in the human gut ecosystem | PRJDB2036 |
| ERP015692 | The_mechanism_of_nutritional_therapy_for_Paediatric_Crohn_s_disease | PRJEB14084 |
| ERP105282 | WGS data from FMT trial for the treatment of recurrent Clostridium difficile infection | PRJEB23524 |
