# Supplementary material for: Microbial imbalance in inflammatory bowel disease patients at different taxonomic levels
Source: Gut Pathog. 2020 Jan 4;12:1. doi: 10.1186/s13099-019-0341-6 (PMC6942256; doi:10.1186/s13099-019-0341-6)
Supplement: Supplementary file 2 — Additional file 2: Figure S1. Phylum level richness in the gut microbiota from our samples (shown in green) compared to the gut microbial species richness obtained across studies, each with more than 100 OTUs (total 81 study), from a variety of conditions (shown in black). [file 13099_2019_341_MOESM2_ESM.docx]

**
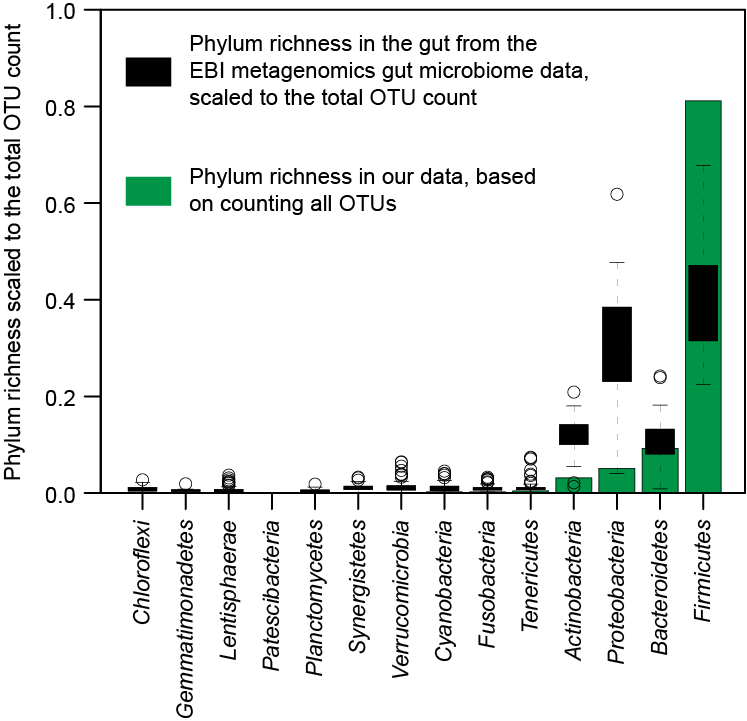
**

**Additional file 2: Figure S1.** Phylum level richness in the gut microbiota from our samples (shown in green) compared to the gut microbial species richness obtained across studies, each with more than 100 OTUs (total 81 study), from a variety of conditions (shown in black).
